# A mutational process signature and genomic alterations associated with outcome and immunogenicity in cancers with brain metastasis

**DOI:** 10.3389/fimmu.2025.1607772

**Published:** 2025-07-30

**Authors:** Wanli Sun, Xueying Wang, Yixin Xu, Yanfeng Ren, Wenjing Zhang, Qinghua Wang, Yingzhi Chong

**Affiliations:** ^1^ Department of Clinical Laboratory, Affiliated Hospital of Shandong Second Medical University, Weifang, Shandong, China; ^2^ Department of Health Statistics, Key Laboratory of Medicine and Health of Shandong Province, School of Public Health, Shandong Second Medical University, Weifang, Shandong, China; ^3^ School of Medical Laboratory, Shandong Second Medical University, Weifang, Shandong, China; ^4^ Department of Epidemiology, School of Public Health, Shandong Second Medical University, Weifang, Shandong, China

**Keywords:** brain metastasis, prognosticators, mutational signatures, molecular subtypes, genomic variations, tumor immunogenicity

## Abstract

**Background:**

Brain metastasis (BM) is one of the common ways of tumor metastasis and has a poor prognosis. This study aims to identify potential biomarkers from the perspective of somatic mutations, providing a basis for the prognosis evaluation and immunogenicity prediction of BM patients.

**Methods:**

This study collected the somatic mutation profiles and clinical information of a total of 421 patients with BM in Memorial Sloan Kettering Cancer Center (MSKCC). Non-negative matrix factorization was employed to extract the mutational process signatures operating in the genome. Consensus clustering analysis was utilized to identify mutation-related molecular subtypes. Through a comprehensive analysis of genomic mutations and copy number variations (CNV), biomarkers associated with outcomes and tumor immunogenicity were screened.

**Results:**

Non-small cell lung cancer, melanoma, and breast cancer were common primary tumors of BM, and these three tumor types exhibited better prognosis compared to other types. This study found that a higher tumor mutation burden (TMB) was significantly associated with a better prognosis of BM. A total of four mutational process signatures were extracted, and among them, a signature featured by C > T mutations and related to DNA damage repair was proven to be linked with an inferior outcome and a lower TMB. Through integrated genomic mutation analysis, *PTPRT* mutation was determined to associate with improved prognosis of BM. More importantly, patients carrying this mutation also harbored a better response to immunotherapy. CNV analysis indicated that *PTEN* deletion and *DUSP4* deletion were respectively associated with poorer and better outcomes in patients with BM.

**Conclusions:**

By integrating the somatic mutation data of patients with BM, this study identified molecular biomarkers related to outcomes and immunogenicity from three perspectives: mutational process signatures, molecular subtypes, and genomic variations. Our findings provide clues for prognosis evaluation in BM patients. They also establish a theoretical basis for predicting immunotherapy efficacy.

## Introduction

Brain metastasis (BM) represents a devastating complication of advanced cancers, occurring in approximately 10–30% of cancer patients (1), with non-small cell lung cancer, melanoma, and breast cancer being the most common primary origins. Despite advancements in systemic therapies, BM patients face dismal prognoses, with median survival often measured in months (2). The blood-brain barrier and immunosuppressive tumor microenvironment further limit therapeutic efficacy (3), highlighting the urgent need for robust biomarkers to guide prognosis prediction and treatment optimization.

Mutational process signatures (4), defined as genome-wide patterns of somatic mutations resulting from specific DNA damage or repair defects (e.g., environmental carcinogens, enzymatic dysregulation, or therapy-induced stress), have emerged as critical tools in cancer prognosis and treatment. For instance, homologous recombination deficiency-associated signature predicts BRCA1/2 mutations and sensitivity to PARP inhibitors (5), while apolipoprotein B mRNA editing enzyme catalytic polypeptide like (APOBEC) activity-linked signature correlates with immune evasion but may enhance responses to immune checkpoint inhibitors (6, 7). Tobacco-related signature is tied to poor lung cancer outcomes and reduced EGFR-targeted therapy efficacy (8). However, the role of mutational signatures in BM remains unexplored, leaving their prognostic and therapeutic implications unknown.

Single-gene mutation and copy number variations (CNV) significantly influence tumor behavior and therapeutic sensitivity. For example, *TP53* mutations are associated with chemotherapy resistance and poor prognosis across cancers (9, 10), demonstrating how driver mutations can dictate therapeutic outcomes. *HER2* amplification predicts trastuzumab efficacy in breast cancer (11), illustrating how CNVs guide targeted therapy selection. *BRAF V600E* mutations confer sensitivity to BRAF/MEK inhibitors but may diminish immunotherapy responses in melanoma (12), highlighting mutation-dependent trade-offs in treatment efficacy. *EGFR*-sensitive mutations predict robust responses to EGFR-TKIs in lung cancer (13), reinforcing the clinical actionability of specific genomic alterations. Similarly, *MYCN* amplification in neuroblastoma drives tumor aggressiveness and chemoresistance (14). Thus, the above evidence suggests that systematically profiling genomic mutations in BM patients could uncover similarly actionable biomarkers to guide prognosis and therapy.

Immunotherapy activates T cells to recognize and attack tumors. Its advantages lie in the persistent anti-tumor effect and the potential to induce immune memory. In BM patients, the combination of PD-1/PD-L1 inhibitors (such as pembrolizumab) and radiotherapy can penetrate the blood-brain barrier and partially relieve the lesions (15). Survival benefits have been observed especially in patients with BM from melanoma or lung cancer [such as the CheckMate 204 study (16)]. However, due to the limitations of the blood-brain barrier on the efficiency of drug delivery and the insufficient infiltration of T cells in the immune microenvironment of BM, the response rate of some patients is low [only 15%-30% (17)].

By integrating somatic mutation profiles and clinical data, this study aims to elucidate molecular markers–mutational process signatures, genomic molecular subtypes, and actionable genomic variations–to guide prognosis assessment and treatment strategies for BM patients.

## Methods

### BM patients and immunotherapy-treated patients

A total of 421 patients with BM tumors, whose data included somatic mutation, CNV information and clinicopathological details (**Supplementary Table S1**), were enrolled in this study from the Memorial Sloan Kettering Cancer Center (MSKCC). These data were sourced from three previously published studies (18–20). The primary tumors of the BM patients included non-small cell lung cancer (NSCLC), melanoma (SKCM), breast cancer (BRCA), and other tumor types with relatively small proportions, amounting to a total of 21 types. Somatic mutation data of all the above-mentioned patients were obtained using the MSK-IMPACT targeted sequencing approach (21). In this study, the Oncotator software was employed to conduct standardized annotation of all the mutation data (22), aiming to eliminate sequencing biases and facilitate subsequent analyses.

**Figure 1:**
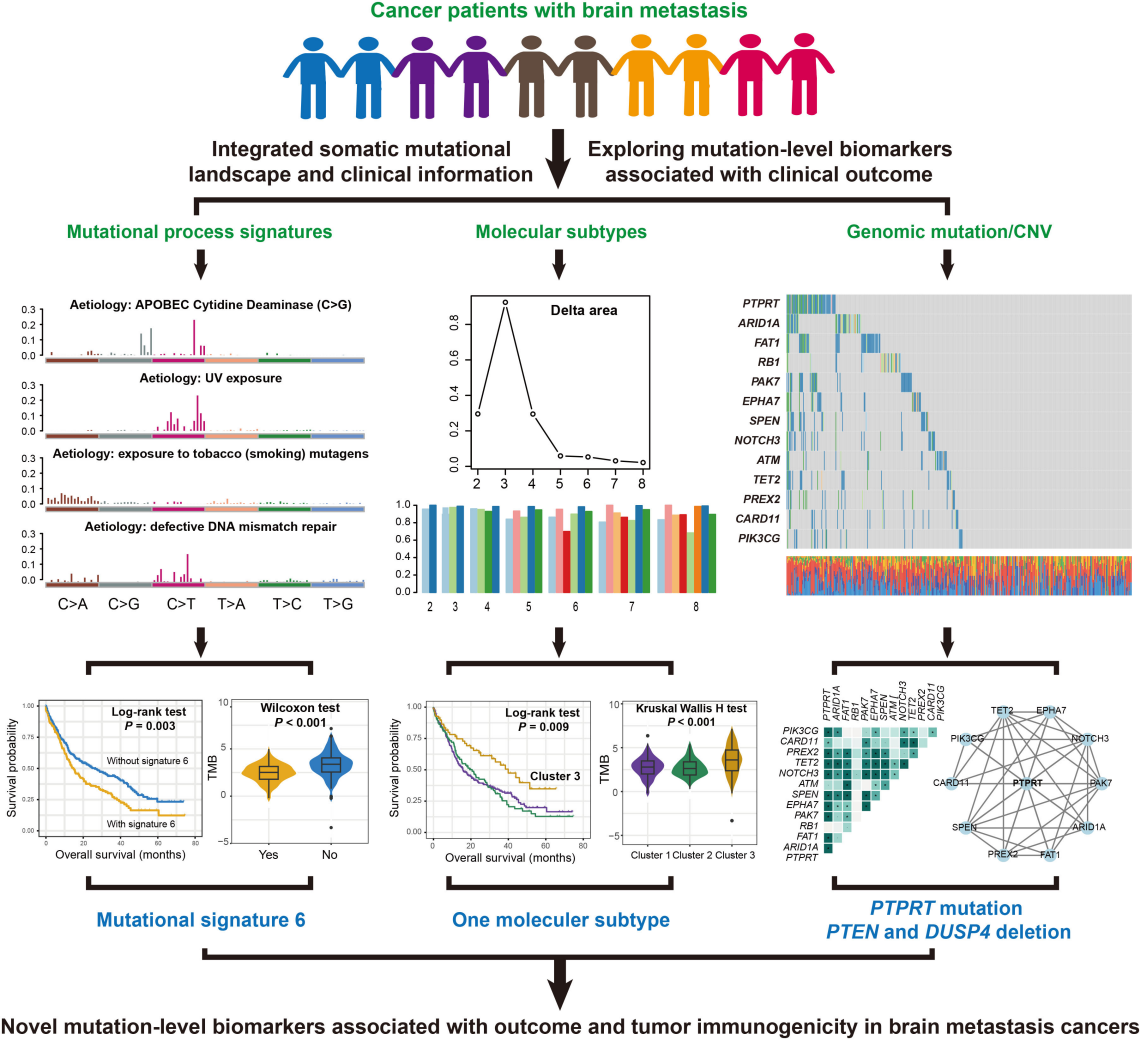
A detailed flowchart presented the identification process of prognostic and immunogenic mutation molecular biomarkers in patients with BM.

This study retrospectively integrated pretreatment whole-exome sequencing (WES) data of somatic mutations, clinical characteristics, and immune checkpoint inhibitor (ICI) treatment follow-up information from 109 NSCLC (23, 24) and 631 SKCM (7, 25–31) patients. NSCLC patients were treated with anti-PD-1 monotherapy or combination immunotherapies. SKCM patients received anti-CTLA-4, anti-PD-1/PD-L1, or combination therapies. Somatic mutation profiles from pretreatment samples were also annotated using the Oncotator. For subsequent analyses, we focused on nonsynonymous mutations encompassing missense mutations, frameshift insertions/deletions (indels), inframe indels, nonsense mutations, and splice site mutations. Detailed clinical characteristics and ICI treatment regimens, including therapeutic responses, were systematically documented in **Supplementary Table S2** (NSCLC patients) and **Supplementary Table S3** (SKCM patients). The detailed workflow of this study was shown in **Figure 1**.

### Mutational process signatures operative in BM cancers

The mutational process signature extraction workflow was performed using non-negative matrix factorization (NMF) algorithm (32). Somatic mutation data of BM patients, formatted as Mutation Annotation Files (MAF), were preprocessed with the R ​maftools package (33) to generate a 96-dimensional trinucleotide context matrix, where rows represented samples and columns corresponded to the 96 possible single-base substitution types within their trinucleotide contexts. Low-frequency mutations (occurring in < 1% of samples) were excluded to minimize noise.

The NMF algorithm, executed via the ​NMF package, decomposed the normalized mutation count matrix into two non-negative matrices: a signature matrix (W, *k* × 96 dimensions, defining mutational patterns) and an exposure matrix (H, sample × *k* dimensions, quantifying signature contributions per sample). The optimal number of signatures (*k*) was determined through cross-validation (10 iterations per *k*) using the ​nmfEstimateRank function, with stability and reconstruction error metrics. Extracted signatures were annotated by cosine similarity matching against the COSMIC v2.0 database (34) via the ​cosmicsig package to identify known etiological processes. In this study, all mutational signatures were defined as binary variables [following the method described in a previous study (35)]: if the activity of a certain mutational signature exceeded 25%, it was considered that this signature was present in the sample.

### Consensus clustering based on the mutational signature activities

Molecular subtyping of BM patients was performed via consensus clustering analysis using the R package ​ConsensusClusterPlus (36). Mutational signature activity data, quantified as the normalized exposure of each signature per sample. Signatures with standard deviation < 0.1 (normalized exposure) were excluded to retain biologically informative patterns, as low-variance signatures may reflect technical noise rather than true molecular heterogeneity. The consensus clustering algorithm was applied with the following parameters: maximum cluster number (*k*= 8), 1000 iterations, resampling 80% of samples per iteration (pItem = 0.8), Pearson correlation distance, and hierarchical clustering with Ward’s linkage. Cluster stability was evaluated by consensus cumulative distribution function (CDF) curves and delta area analysis, with the optimal *k* determined by the plateau in consensus index scores and minimal inter-cluster cross-talk. Final molecular subtypes were defined based on the highest cluster consensus (> 0.9).

### Co-occurrence and mutual exclusivity of BM prognostically mutated genes

Somatic mutation co-occurrence and mutual exclusivity analyses were performed using the R package ​maftools (33). For BM patients with available MAF, recurrently mutated genes (altered in ≥ 5% of samples) were first identified, followed by filtering low-penetrance variants (germline polymorphisms excluded by dbSNP v155). Gene pairs exhibiting significant co-occurrence or mutual exclusivity were assessed using the somaticInteractions() function, which applies Fisher’s exact test (two-tailed) with Benjamini-Hochberg correction (FDR < 0.1) to evaluate deviation from random mutation patterns. Prognostic relevance was determined by integrating survival data into a Cox proportional hazards model, where gene pairs with significant interaction terms (*P* < 0.05) were prioritized. Heatmaps of co-occurrence/exclusivity networks were generated using the ​ComplexHeatmap package (37).

### Statistical analysis and visualization

Statistical analyses and data visualization were performed using R software. Pie charts, box plots, and heatmaps were constructed with the ggplot2 package. Lollipop plots visualizing amino acid alteration patterns induced by gene mutations were generated using the maftools package. Survival curves were plotted via Kaplan-Meier analysis, and between-group differences were evaluated by the Log-rank test. Multivariable logistic regression and Cox regression models incorporating confounding factors were implemented with the forestmodel package. In the multivariate model, samples with missing values were deleted. We performed Schoenfeld residual analysis (using the survival R package) to validate that the Cox model proportional hazards (PH) assumptions were met. Wilcoxon rank-sum test and Kruskal-Wallis H test were applied to compare non-normally distributed variables across two and three groups, respectively. Two-sided *P* values less than 0.05 were deemed statistically significant.

## Results

### Primary tumor subtypes and TMB linked with prognosis in BM patients

**Figure 2:**
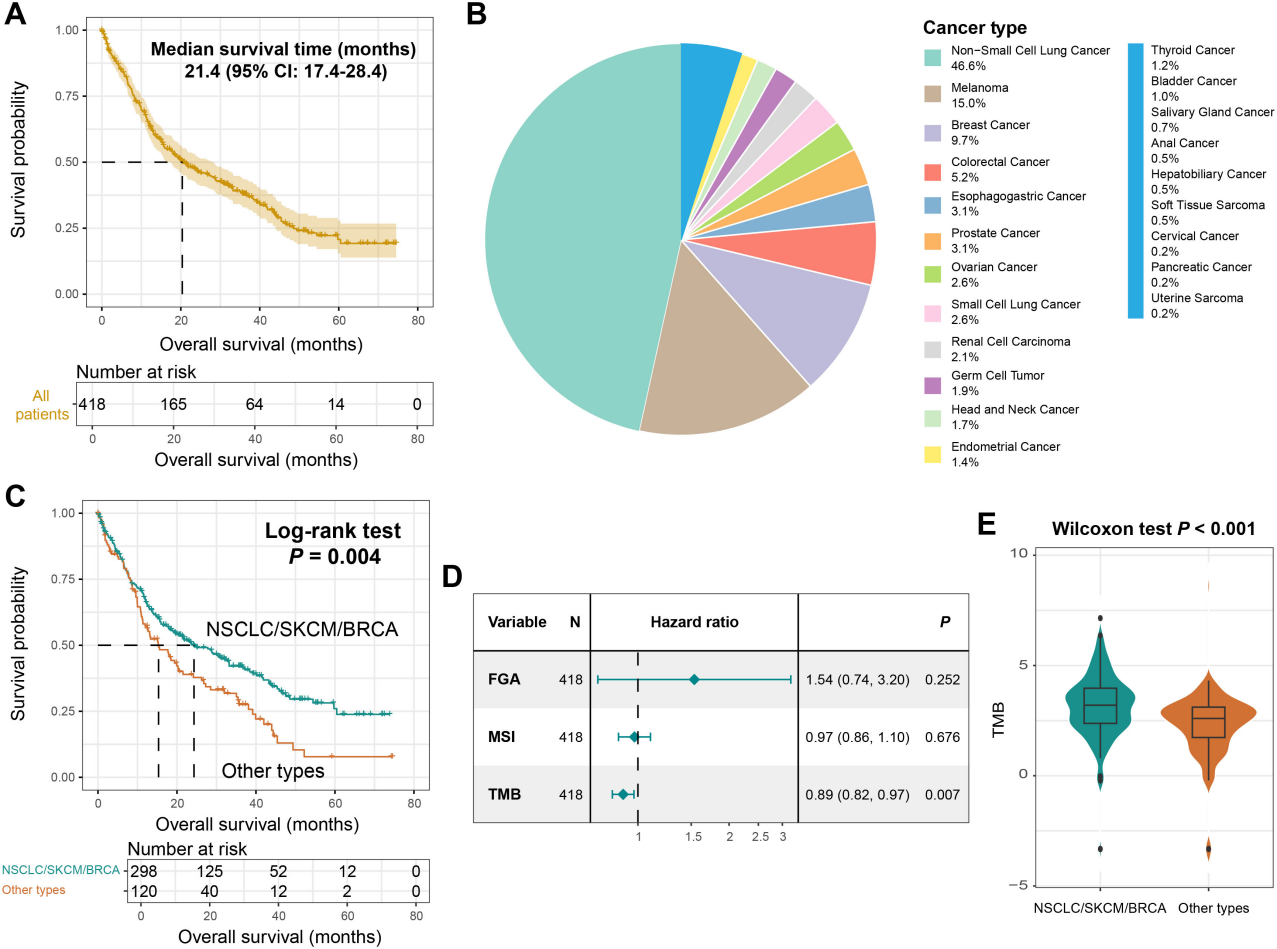
The primary tumor types of BM and TMB were associated with prognosis. **(A)** Survival curves of all BM patients included in this study. **(B)** A pie chart showing the primary tumors of BM patients. **(C)** Kaplan-Meier survival curves of NSCLC/SKCM/BRCA patients and other patients. **(D)** A Cox regression model of the relationship between FGA, MSI, TMB and BM prognosis. **(E)** Distinct TMB distributions between NSCLC/SKCM/BRCA patients and other patients.

A total of 421 tumor patients with BM and 5882 somatic mutations were included in this study. The median overall survival (OS) for the entire cohort was 21.4 (95% CI: 17.4–28.4) months (**Figure 2A**). The mutational profile of patients with BM is predominantly characterized by C > T single-nucleotide polymorphism missense mutations (**Supplementary Figure S1**). Primary tumors of BM encompassed 21 histological subtypes (**Figure 2B**), with NSCLC (46.6%), SKCM (15.0%), and BRCA (9.7%) representing the three most prevalent subtypes, followed by colorectal cancer, esophagogastric cancer, and rare tumor types (e.g., uterine sarcoma, pancreatic cancer, and cervical cancer).

Kaplan-Meier analysis revealed that patients with BM originating from NSCLC, SKCM, or BRCA exhibited significantly prolonged survival compared to those with other primary tumors (Log-rank test, *P* = 0.004; **Figure 2C**, **Supplementary Figure S2**). This study further explored the prognostic roles of the indicators related to tumor genomic instability, namely fraction genome altered (FGA), microsatellite instability (MSI), and tumor mutational burden (TMB). The results demonstrated that a higher TMB was associated with a better prognosis (HR: 0.89, 95% CI: 0.82-0.97, *P* = 0.007, **Figure 2D**). Meanwhile, we found that BM patients originating from NSCLC, SKCM, or BRCA harbored a significantly elevated TMB (Wilcoxon rank-sum test, *P* < 0.001; **Figure 2E**). These findings underscore TMB and tumor origin as dual determinants of BM outcomes.

### A mutational process signature linked with BM outcome and immunogenicity

**Figure 3:**
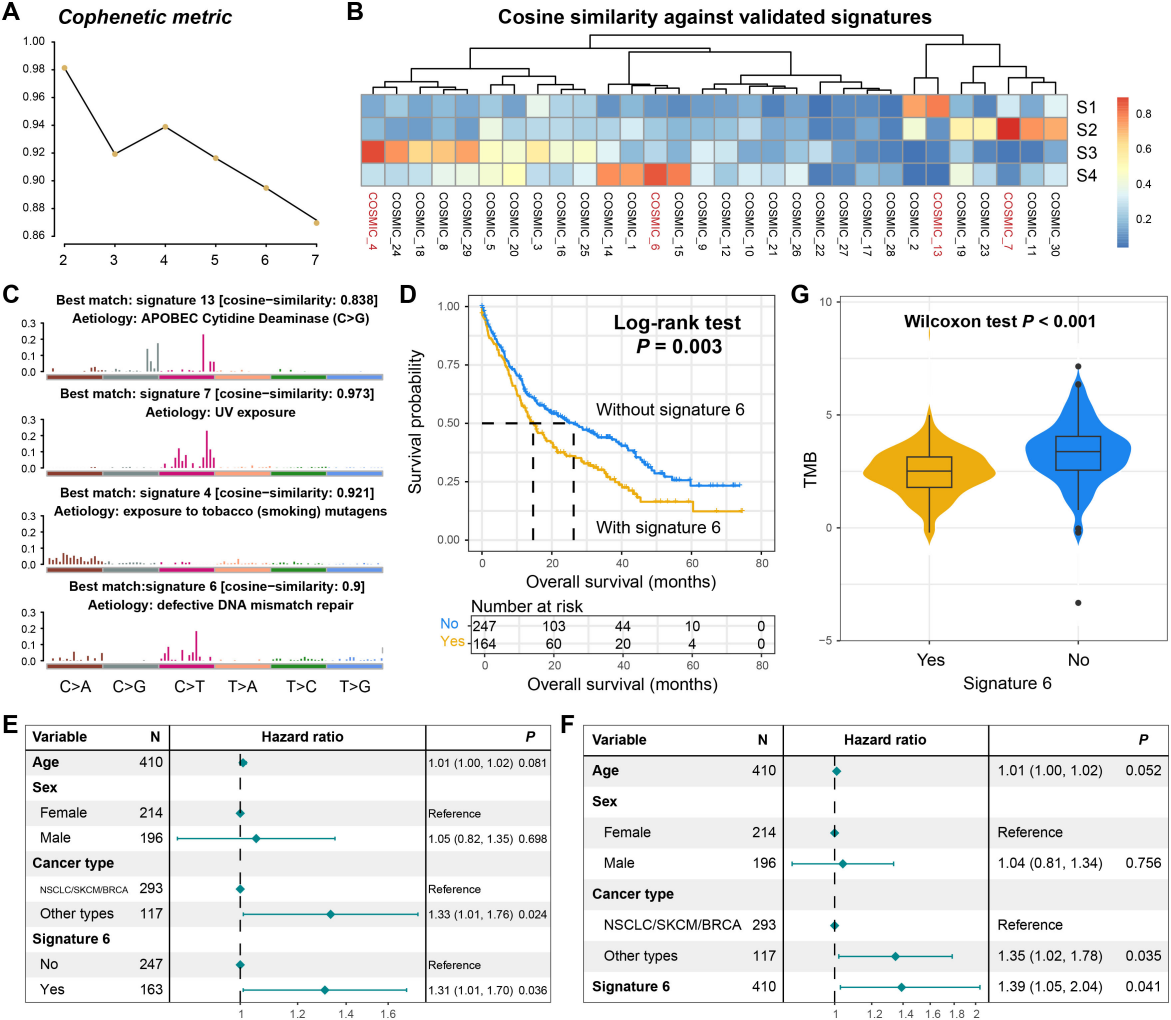
Mutational process signatures operative in the BM genome. **(A)** The cophenetic metric plot for determining the optimal number of mutational signatures. **(B)** The correlation heatmap between the four extracted mutational signatures and the annotated signatures in the COSMIC database. **(C)** The detailed mutation patterns of the four extracted mutational signatures. **(D)** The survival curves of BM patients with and without signature 6. **(E)** The multivariate adjusted Cox regression model of the relationship between signature 6 as a binary variable and prognosis. **(F)** The multivariate adjusted Cox regression model of the relationship between signature 6 as a continuous variable and prognosis. **(G)** The distribution of TMB in BM patients with and without signature 6.

Based on the NMF algorithm applied to the somatic mutation matrix of BM patients, four distinct mutational process signatures were extracted (**Figure 3A**). Through cross-referencing with the COSMIC database of annotated mutational signatures, these extracted signatures were systematically classified as COSMIC signatures 4, 6, 7, and 13 according to standardized nomenclature (**Figure 3B**). Their characteristic mutational patterns were illustrated in **Figure 3C** and detailed mutational activities were shown in **Supplementary Table S4**. Signature 4 has been implicated in tobacco exposure-related carcinogenesis, while signature 6 is associated with defective DNA damage repair mechanisms. Signature 7 was identified as the ultraviolet light-induced mutational pattern, and signature 13 was linked to APOBEC enzyme activity. Notably, the APOBEC-associated mutational signature demonstrated widespread prevalence across multiple tumor types. In this BM cohort, patients carrying this signature exhibited significantly elevated mutation burden (**Supplementary Figure S3A**) and tCw motif burden (a specific nucleotide context preferentially targeted by APOBEC enzymes) (**Supplementary Figure S3B**). Furthermore, the presence of APOBEC signature showed significant correlations with mutations in multiple cancer-related genes (**Supplementary Figure S3C**).

We subsequently investigated the prognostic relevance of the four identified mutational signatures. Survival analysis revealed no significant association between the presence of signatures 4, 7, or 13 and clinical outcomes in BM patients (**Supplementary Figures S4A–S4C**). Strikingly, patients harboring signature 6 (DNA damage repair associated) exhibited significantly worse overall survival (Log-rank test, *P* = 0.003; **Figure 3D**). This association remained robust in a multivariable Cox regression model adjusted for age, sex, and tumor type (HR: 1.31, 95% CI: 1.01–1.70, *P* = 0.036, **Figure 3E**). Moreover, when signature 6 was analyzed as a continuous variable in the Cox regression model, its correlation with the inferior prognosis retained statistical significance (HR: 1.39, 95% CI: 1.05–2.04, *P* = 0.041; **Figure 3F**). Subsequently, we conducted a stratified analysis of the prognostic ability of signature 6 based on the primary tumor type. The results showed that in NSCLC/SKCM/BRCA, patients carrying signature 6 still exhibited poorer prognosis (Log-rank test, *P* = 0.047; **Supplementary Figure S5A**). However, in other tumor types with a smaller proportion, there was no significant survival difference between patients with and without this mutational signature (Log-rank test, *P* = 0.238; **Supplementary Figure S5B**), but a consistent trend was observed.

Intriguingly, BM patients carrying signature 6 demonstrated significantly reduced TMB compared to non-carriers (Wilcoxon rank-sum test, *P* < 0.001, **Figure 3G**). Emerging evidence suggests that TMB serves as a surrogate marker for tumor immunogenicity (38, 39), which critically influences antitumor immune responses. Our findings propose a potential mechanistic link wherein signature 6 may potentially compromise prognosis through diminished immunogenicity, though further functional validation is warranted.

### Mutational activity-derived molecular subtypes

**Figure 4:**
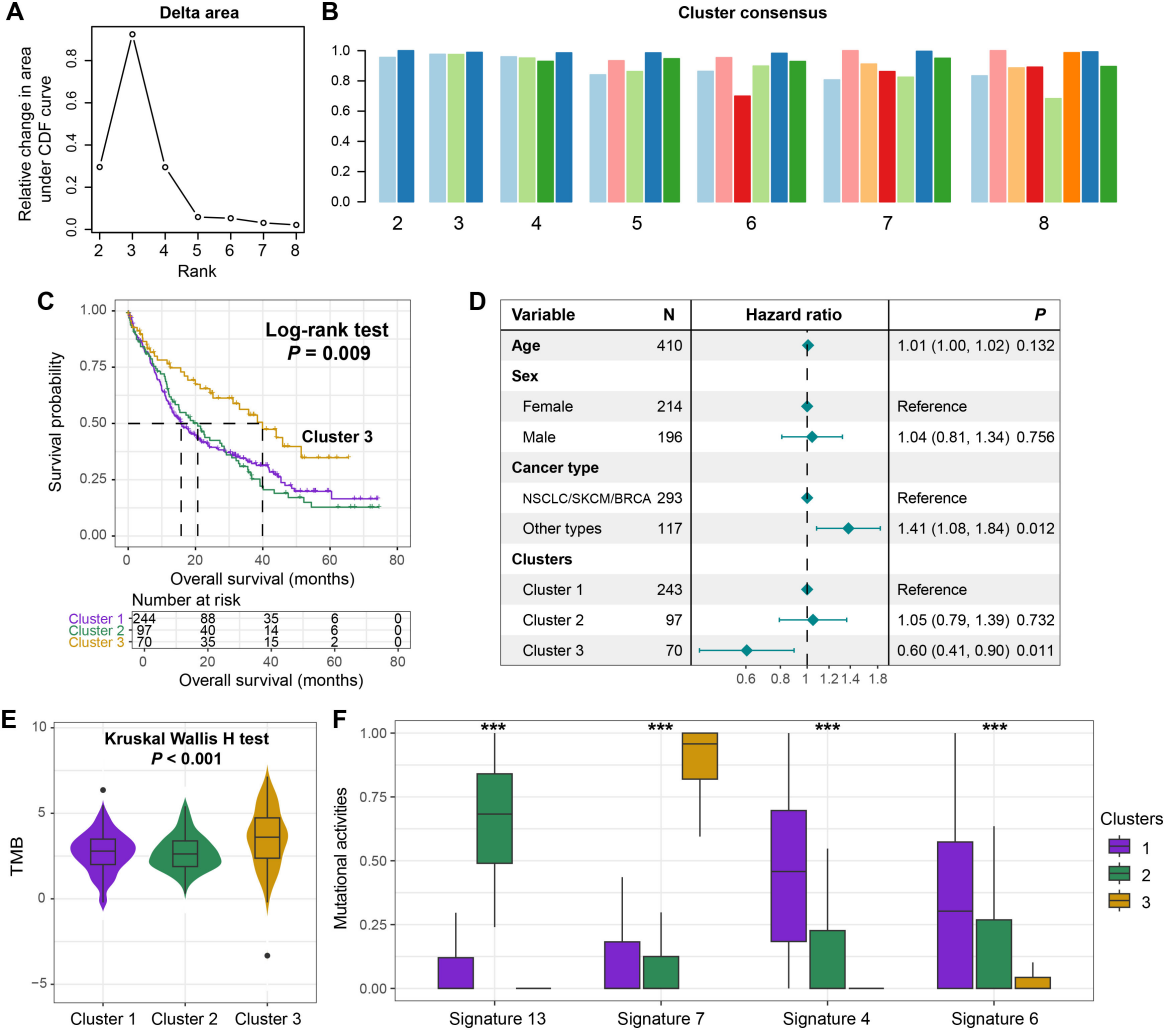
Identification of prognostic molecular subtypes driven by mutational activity. **(A)** The area graph under the clustering CDF curve. **(B)** The clustering consistency when the number of clusters ranged from 2 to 8. **(C)** The survival curves of the three clusters obtained through clustering based on the activity of mutational signatures. **(D)** A multivariate Cox regression model incorporating age, sex, and tumor type to elucidate the association between the clusters and the prognosis of BM. **(E)** The distribution of TMB among the three distinct clusters. **(F)** The different distributions of the activities of the four extracted mutational signatures among the three clusters. ***P < 0.001.

While most molecular classification studies in oncology rely on transcriptomic profiling, such approaches are inherently challenged by high-dimensional data complexity and technical noise. This study innovatively leveraged the activity profiles of extracted mutational signatures to investigate potential molecular subtypes in BM patients through consensus clustering analysis (**Supplementary Figure S6A**). The clustering optimization analysis revealed maximal consensus stability with three clusters, as evidenced by the inflection point (maximum slope) in the CDF curve (**Figure 4A**). Clustering consensus of three clusters was further validated by the plateauing consensus index in the clustering bar plot (**Figure 4B**), with detailed clustering heatmap visualizing in **Supplementary Figure S6B**.

Kaplan-Meier survival analysis demonstrated that cluster 3 exhibited significantly improved survival compared to clusters 1 and 2 (Log-rank test, *P* = 0.009, **Figure 4C**). This prognostic advantage persisted in the multivariable Cox regression model adjusted for clinicopathological covariates (HR = 0.60, 95% CI: 0.41–0.90, *P* = 0.011, **Figure 4D**). Subsequently, we conducted a stratified analysis of the prognostic ability of identified clusters based on the primary tumor type. The results showed that in NSCLC/SKCM/BRCA, patients from cluster 3 still exhibited favorable prognosis compared with cluster 1 and 2 (Log-rank test, *P* = 0.038, **Supplementary Figure S7A**). However, in other tumor types with a smaller proportion, there was no significant survival difference between identified three clusters (Log-rank test, *P* = 0.382, **Supplementary Figure S7B**), but a consistent trend was observed.

BM patients from the cluster 3 also displayed an elevated TMB relative to other clusters (Kruskal-Wallis H test, *P* < 0.001; **Figure 4E**), suggesting enhanced tumor immunogenicity as a potential mechanism underlying its favorable prognosis. Furthermore, the four mutational signatures exhibited differential activity distribution across clusters. Cluster 3 was characterized by heightened signature 7 activity alongside suppressed activities of signatures 4, 6, and 13 (**Figure 4F**), indicating distinct biological etiologies among molecular subgroups. This signature activity distribution provides mechanistic insights into the observed survival disparity and TMB heterogeneity.

### Recurrently prognostically mutated genes in BM patients

**Figure 5:**
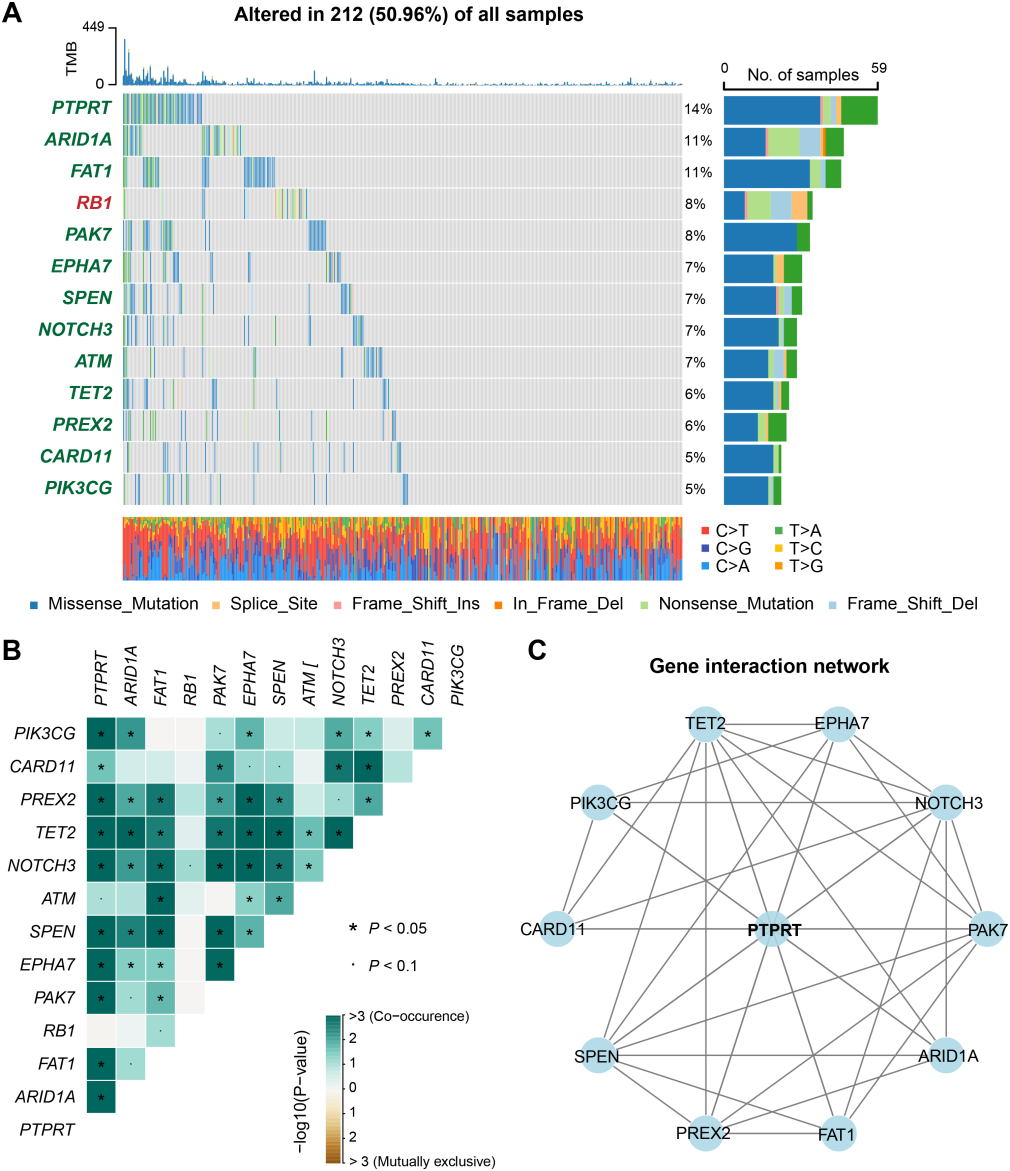
Discovery of clinically actionable genomic mutations linked with BM outcome. **(A)** A waterfall plot showed the mutation patterns of recurrently mutated genes associated with either better or worse prognosis of BM. Genes marked in red indicated that their mutations were associated with a poor prognosis, while those marked in green indicated an association with a better prognosis. **(B)** A heatmap of the co-occurrence or mutual exclusivity of genes related to the prognosis of BM. **(C)** An interaction network of genes related to the prognosis of BM.

We systematically investigated the prognostic implications of all somatic mutations in BM patients. Initial profiling revealed high-frequency mutations (≥ 5% mutation rate) in key driver genes including *TP53*, *KRAS*, *EGFR*, *PTPRT*, *PTPRD*, *APC*, et.al., with *TP53* (58% of patients) emerging as the predominant mutational driver in BM (**Supplementary Figure S8**). Subsequently, Cox regression identified 12 recurrently mutated genes significantly associated with clinical outcomes (*P* < 0.05), namely *PTPRT*, *ARID1A*, *FAT1*, *PAK7*, *EPHA7*, *SPEN*, *NOTCH3*, *ATM*, *TET2*, *PREX2*, *CARD11*, and *PIK3CG* (**Figure 5A**). Unexpectedly, while *RB1* mutations correlated with inferior prognosis (**Supplementary Figure S9**, **Figure 5A**), all other prognostic mutations demonstrated protective effects (**Figure 5A**).

Functional enrichment analysis mapped these prognostic genes to critical oncogenic pathways including NOTCH signaling, HIPPO signaling, and cell cycle regulation (**Supplementary Figure S10**), with their potential therapeutic targetability stratified by druggable categories in **Supplementary Figure S11**. Furthermore, we explored the co-occurrence and mutual exclusivity among the prognostically mutated genes (**Figure 5B**, **Supplementary Table S5**). The results revealed significant co-occurrence patterns between *PTPRT* and multiple prognostic genes, with the highest interaction frequency (**Figure 5C**), suggesting a central role for *PTPRT* mutations in modulating BM outcomes through synergistic genetic interactions.

### *PTPRT* mutation for BM outcome and immunotherapeutic implications

**Figure 6:**
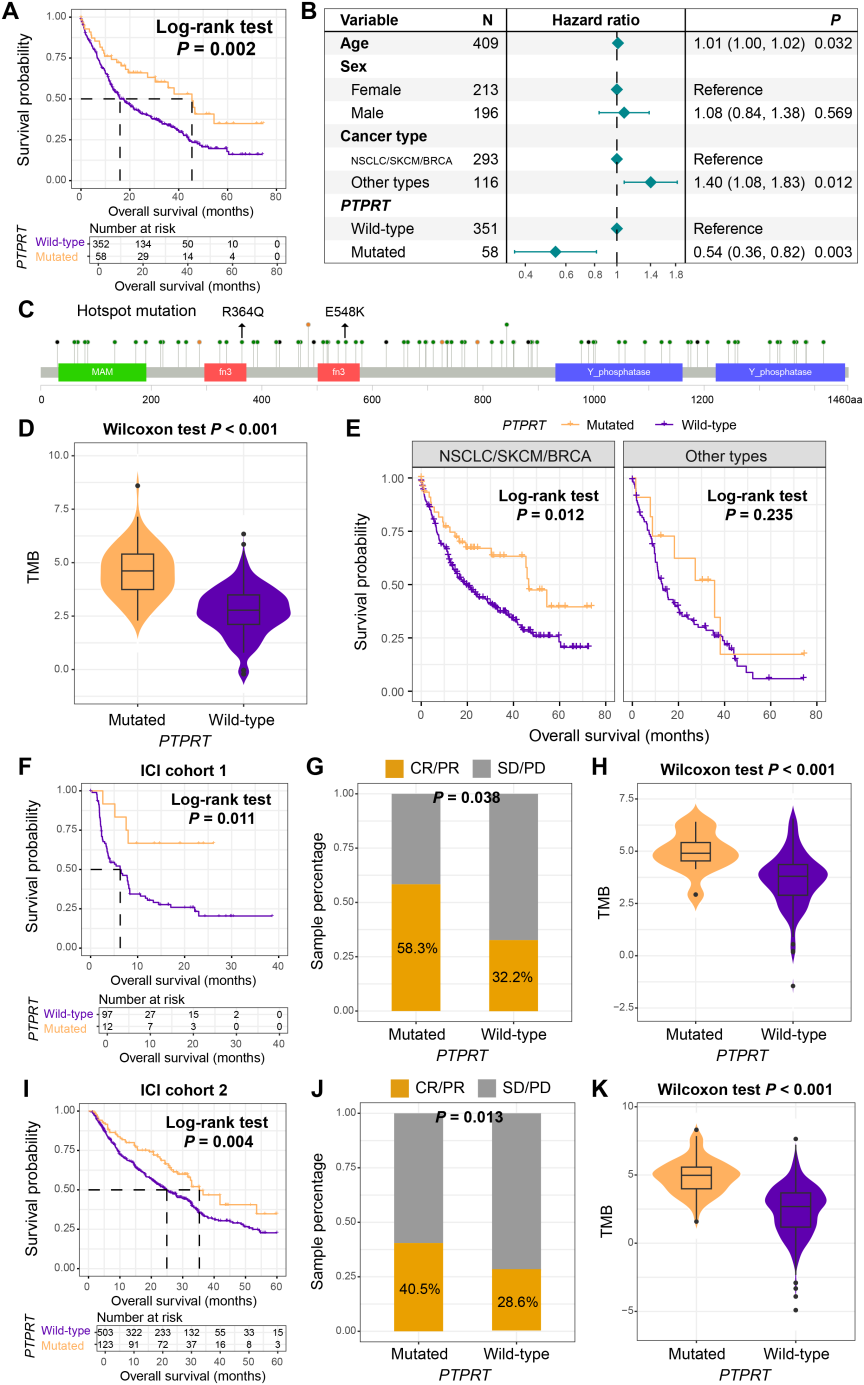
*PTPRT* mutation as a prognostic and immunotherapeutic biomarker. **(A)** Kaplan-Meier survival curves of BM patients with and without *PTPRT* mutation. **(B)** A multivariate Cox regression model of the association between *PTPRT* mutation and the prognosis of BM. **(C)** Detailed information on the amino acid changes caused by *PTPRT* mutations. **(D)** The differential distribution of TMB between patients with *PTPRT* mutation and those with the wild-type *PTPRT*. **(E)** The prognostic role of *PTPRT* mutation in different primary tumor types of BM. Among NSCLC patients who received ICI treatment, the relationships between *PTPRT* mutation and **(F)** prognosis, **(G)** treatment response rate, and **(H)** TMB. Among SKCM patients who received ICI treatment, the relationships between *PTPRT* mutation and **(I)** prognosis, **(J)** treatment response rate, and **(K)** TMB.

Previous analyses have indicated that *PTPRT* played a crucial role in the mutation interaction network of BM. In both univariate survival analysis (Log-rank test, *P* = 0.002, **Figure 6A**) and multivariable Cox regression model (HR = 0.54, 95% CI: 0.36–0.82, *P* = 0.003, **Figure 6B**), we found that patients with *PTPRT* mutations had significantly improved survival outcomes. The lollipop plot presented the detailed amino acid changes caused by *PTPRT* mutations, with *R364Q* and *E548K* being the hotspot mutations (**Figure 6C**). Further analysis demonstrated that BM patients carrying *PTPRT* mutations harbored significantly elevated TMB levels (Wilcoxon rank-sum test, *P* < 0.001, **Figure 6D**).

Interestingly, stratified survival analysis by tumor origin revealed preserved prognostic advantage of *PTPRT* mutations in NSCLC/SKCM/BRCA (Log-rank test, *P* = 0.012, **Figure 6E**), but not in other subtypes. Considering that NSCLC and SKCM are two common tumor types for current immunotherapy, and the TMB was significantly increased in patients with *PTPRT* mutations, we hypothesized whether this mutation could evaluate the efficacy of immunotherapy. Based on a cohort of 109 NSCLC patients who received ICI treatment (ICI cohort 1), this study found that patients with *PTPRT* mutations exhibited better ICI prognosis (Log-rank test, *P* = 0.011, **Figure 6F**). Meanwhile, the treatment response rate (the proportion of CR and PR) was significantly increased (mutant vs. wild-type: 58.3% vs. 32.2%, Fisher exact test, *P* = 0.038, **Figure 6G**), and TMB was also enriched in the mutant patients (Wilcoxon rank-sum test, *P* < 0.001, **Figure 6H**). In the validation cohort of 631 ICI-treated patients with SKCM (ICI cohort 2), improved survival outcomes (**Figure 6I**), increased response rates (**Figure 6J**), and elevated TMB (**Figure 6K**) were also observed in *PTPRT* mutant patients. *PTPRT* mutations improved survival and enhanced immunotherapy response in NSCLC and SKCM patients, which suggests that *PTPRT* may serve as a pan-cancer biomarker for immune checkpoint inhibitor efficacy.

### CNV-driven prognostic biomarkers

**Figure 7:**
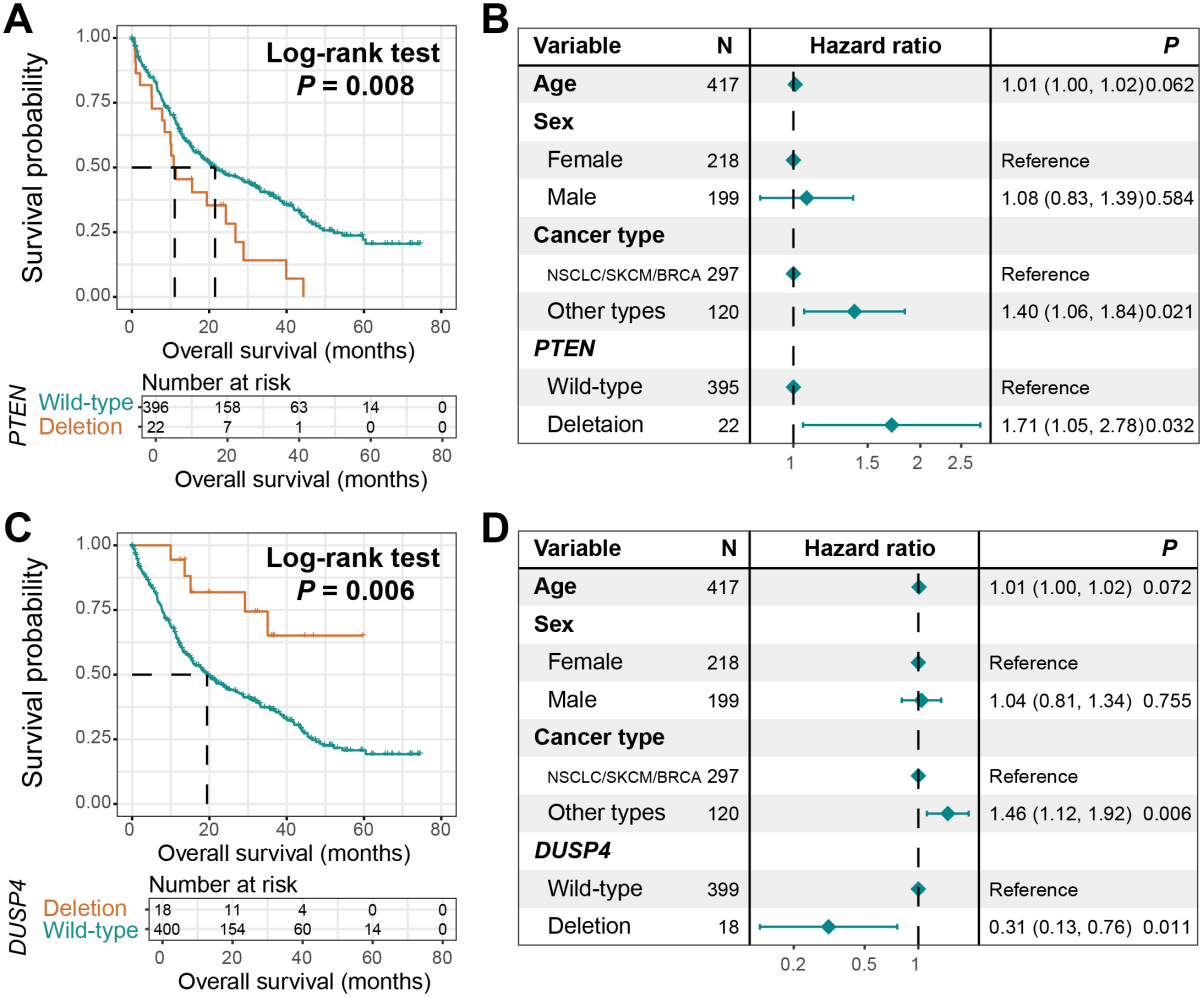
CNV-driven prognostic markers. **(A)** Survival curves of BM patients with *PTEN* deletion and those with wild-type *PTEN*. **(B)** A Cox adjusted regression model of the relationship between *PTEN* deletion and the prognosis of BM, incorporating age, sex, and tumor type. **(C)** Survival curves of BM patients with *DUSP4* deletion and those with wild-type *DUSP4*. **(D)** A Cox adjusted regression model of the relationship between *DUSP4* deletion and the prognosis of BM, incorporating age, sex, and tumor type.

We systematically investigated the associations between copy number variations (CNVs) of all included genes and clinical outcomes in BM patients. Two critical genes, *PTEN* and *DUSP4*, were identified through this screening, both exhibiting recurrent copy number deletion patterns. Survival analysis revealed that BM patients harboring *PTEN* deletions demonstrated significantly reduced overall survival (Log-rank test, *P* = 0.008, **Figure 7A**). This association retained statistical significance in multivariable Cox regression models after adjusting for key prognostic confounders including age, sex, and primary tumor histology (HR: 1.71, 95% CI: 1.05–2.78, *P* = 0.032, **Figure 7B**).

Conversely, *DUSP4* deletions conferred a protective effect, with carriers showing significantly prolonged survival compared to non-deletion controls (Log-rank test, *P* = 0.006, **Figure 7C**). The favorable prognostic value of *DUSP4* deletions remained robust upon multivariable adjustment (HR: 0.31, 95% CI: 0.13–0.76, *P* = 0.011, **Figure 7D**).

## Discussion

This study systematically characterized the somatic mutation landscape of BM through mutational process signature analysis, molecular subtyping, and genomic variation profiling. Defective DNA damage repair-associated signature 6 linked to poor prognosis and reduced immunogenicity. Consensus clustering revealed a TMB-high, immunogenicity-enriched subtype with superior survival, while integrated genomic variation analysis highlighted *PTPRT* mutations, *PTEN* and *DUSP4* deletions as novel biomarkers for prognosis or immunotherapy response prediction. These findings collectively provided a multi-dimensional framework for understanding BM prognostic biomarkers.

The study identified four distinct mutational process signatures in BM, among which signature 6 (linked to defective DNA damage repair) was significantly associated with poor prognosis. This finding aligned with previous reports demonstrating that impaired DNA repair processes promote genomic instability and therapeutic resistance in advanced cancers (40, 41). However, the inverse correlation between signature 6 activity and TMB suggested a paradoxical relationship, where defective repair mechanisms might paradoxically reduce immunogenic mutations, thereby diminishing anti-tumor immune responses. This observation highlighted the complexity of mutational processes in shaping both tumor evolution and immune recognition, underscoring the need to evaluate mutational signatures as independent prognostic markers beyond TMB alone.

Consensus clustering based on mutational signature activities stratified BM patients into three subtypes, with cluster 3 (enriched in UV-associated signature 7 and high TMB) demonstrating superior survival outcomes. This subtype-specific survival advantage likely stemmed from enhanced immunogenicity, as elevated TMB has been widely linked to improved responses to immunotherapies (42, 43). The dominance of UV-related mutations in cluster 3 mirrored findings in primary melanoma (44), suggesting shared etiological mechanisms between primary tumors and their brain metastases. These results emphasized the clinical utility of mutational signature-driven subtyping for risk stratification and immunotherapy candidate selection in BM patients.

The discovery of *PTPRT* mutation as a favorable prognostic factor in BM expanded its known tumor-suppressive roles in primary cancers. Patients harboring *PTPRT* mutations exhibited prolonged survival and heightened TMB, potentially due to its regulatory effects on STAT3-mediated oncogenic signaling and immune modulation (45, 46). Importantly, the association between *PTPRT* mutations and improved ICI responses in both NSCLC and melanoma cohorts suggested its pan-cancer predictive value. This finding paralleled prior studies linking phosphatase gene mutations [e.g., *PTPRD* (47, 48)] to enhanced anti-tumor immunity, proposing *PTPRT* as a mechanistically relevant biomarker for guiding immunotherapy in BM. Further investigations into its functional interplay with JAK-STAT pathways could elucidate its role in shaping the tumor-immune microenvironment. However, due to the unavailability of data, we were unable to analyze the association between *PTPRT* mutations and the efficacy of immunotherapy in other tumor types. Therefore, prospective cohorts or mechanistic experiments are needed to validate the relevant results.

The contrasting prognostic impacts of *PTEN* and *DUSP4* deletions revealed critical pathway dependencies in BM progression. *PTEN* loss, a known driver of PI3K-AKT pathway hyperactivation (49), correlated with aggressive clinical behavior, consistent with its role in promoting metastasis and immune evasion across solid tumors. Conversely, *DUSP4* deletion conferred a survival advantage, potentially through dampened ERK/MAPK signaling (50), which has been implicated in blood-brain barrier penetration and metastatic colonization (51, 52). These findings underscored the therapeutic potential of targeting PI3K-AKT in *PTEN*-deficient BM and leveraging MAPK inhibitors in *DUSP4*-deleted cases, providing a rationale for genotype-guided therapy in BM patients.

We acknowledge that while MSK-IMPACT’s targeted sequencing provides clinically actionable data on coding mutations (21), it may miss non-coding variants (e.g., regulatory regions) and structural variations relevant to BM pathogenesis. To address this, we propose future studies employing whole-genome sequencing (WGS) or whole-exome sequencing (WES) with non-coding region capture [e.g., SureSelect XT HS (53)] to comprehensively characterize BM genomes, particularly for identifying non-coding drivers and structural variants that may co-occur with mutational signatures like Signature 6. Regarding biological validation, while COSMIC annotations provide mechanistic hypotheses (e.g., Signature 6 ≈ defective DNA repair), we agree BM-specific validation is needed through CRISPR-engineered BM cell lines, prospective clinical correlation with PARPi sensitivity (54), and spatial transcriptomics to map signature activity within tumor niches.

This study utilized retrospective datasets, which may introduce selection bias due to uneven representation of treatment lines, PD-L1 status, or other unmeasured confounders (e.g., prior therapies, comorbidities). While we adjusted for key clinical variables (age, sex, tumor type), future prospective studies with standardized data collection are needed to validate our findings. Besides, in the stratified analyses of our study, we found that both mutational signature 6, cluster 3, and *PTPRT* mutation maintained their original prognostic capabilities in NSCLC/SKCM/BRCA patients, but failed to reach statistical significance in other primary tumor types with a smaller proportion (~30%). We believe that the smaller sample size is the cause of the lack of statistical significance. Therefore, larger sample size cohorts or external validation are needed in the future to verify our results and ensure the universality of application to all brain metastasis patients.

Our study has several limitations. First, the inclusion of BM patients from diverse primary tumors (21 cancer types) may introduce biological confounding effects, such as variations in metastatic tropism or immune microenvironment, potentially limiting the generalizability of pan-BM biomarkers. Second, the targeted sequencing approach limited the detection of non-coding and structural variants that could contribute to BM pathogenesis. Future work should validate key findings (e.g., molecular clusters, *PTPRT* mutation) in disease-specific BM cohorts (e.g., NSCLC-BM only), integrate multi-omics approaches (WGS, epigenomics) to capture non-coding drivers, and functionally elucidate the mechanisms linking biomarkers like *DUSP4* loss to enhanced immunogenicity.

This study established mutational process signatures, molecular subtypes, and actionable genomic alterations as critical indicators for BM tumor patients risk stratification. Future validation through prospective trials and functional studies will be essential to translate these discoveries into precision therapies for BM patients.

## Data Availability

The original contributions presented in the study are included in the article/**Supplementary Material**. Further inquiries can be directed to the corresponding authors.

## References

[1] Fares J Petrosyan E Dmello C Lukas RV Stupp R Lesniak MS. Rethinking metastatic brain cancer as a CNS disease. Lancet Oncol. (2025) 26:e111–21., PMID: 39914421 10.1016/S1470-2045(24)00430-3

[2] Sperduto PW Mesko S Li J Cagney D Aizer A Lin NU. Survival in Patients With Brain Metastases: Summary Report on the Updated Diagnosis-Specific Graded Prognostic Assessment and Definition of the Eligibility Quotient. J Clin Oncol. (2020) 38:3773–84., PMID: 32931399 10.1200/JCO.20.01255 PMC7655019

[3] Fecci PE Champion CD Hoj J McKernan CM Goodwin CR Kirkpatrick JP. The evolving modern management of brain metastasis. Clin Cancer Res. (2019) 25:6570–80., PMID: 31213459 10.1158/1078-0432.CCR-18-1624 PMC8258430

[4] Koh G Degasperi A Zou X Momen S Nik-Zainal S. Mutational signatures: emerging concepts, caveats and clinical applications. Nat Rev Cancer. (2021) 21:619–37., PMID: 34316057 10.1038/s41568-021-00377-7

[5] Yndestad S Engebrethsen C Herencia-Ropero A Nikolaienko O Vintermyr OK Lillestol RK. Homologous Recombination Deficiency Across Subtypes of Primary Breast Cancer. JCO Precis Oncol. (2023) 7:e2300338., PMID: 38039432 10.1200/PO.23.00338 PMC10703128

[6] Wang S Jia M He Z Liu XS. APOBEC3B and APOBEC mutational signature as potential predictive markers for immunotherapy response in non-small cell lung cancer. Oncogene. (2018) 37:3924–36., PMID: 29695832 10.1038/s41388-018-0245-9 PMC6053356

[7] Miao D Margolis CA Vokes NI Liu D Taylor-Weiner A Wankowicz SM. Genomic correlates of response to immune checkpoint blockade in microsatellite-stable solid tumors. Nat Genet. (2018) 50:1271–81., PMID: 30150660 10.1038/s41588-018-0200-2 PMC6119118

[8] Adler N Bahcheli AT Cheng KCL Al-Zahrani KN Slobodyanyuk M Pellegrina D. Mutational processes of tobacco smoking and APOBEC activity generate protein-truncating mutations in cancer genomes. Sci Adv. (2023) 9:eadh3083., PMID: 37922356 10.1126/sciadv.adh3083 PMC10624356

[9] Daver NG Iqbal S Renard C Chan RJ Hasegawa K Hu H. Treatment outcomes for newly diagnosed, treatment-naive TP53-mutated acute myeloid leukemia: a systematic review and meta-analysis. J Hematol Oncol. (2023) 16:19., PMID: 36879351 10.1186/s13045-023-01417-5 PMC9990239

[10] Vokes NI Chambers E Nguyen T Coolidge A Lydon CA Le X. Concurrent TP53 Mutations Facilitate Resistance Evolution in EGFR-Mutant Lung Adenocarcinoma. J Thorac Oncol. (2022) 17:779–92., PMID: 35331964 10.1016/j.jtho.2022.02.011 PMC10478031

[11] Antolin S Garcia-Caballero L Reboredo C Molina A Mosquera J Vazquez-Boquete A. Is there a correlation between HER2 gene amplification level and response to neoadjuvant treatment with trastuzumab and chemotherapy in HER2-positive breast cancer? Virchows Arch. (2021) 479:853–7., PMID: 33934230 10.1007/s00428-021-03104-7

[12] Dummer R Ascierto PA Gogas HJ Arance A Mandala M Liszkay G. Encorafenib plus binimetinib versus vemurafenib or encorafenib in patients with BRAF-mutant melanoma (COLUMBUS): a multicentre, open-label, randomised phase 3 trial. Lancet Oncol. (2018) 19:603–15. 10.1016/S1470-2045(18)30142-6 29573941

[13] Ding W Yang P Zhao X Wang X Liu H Su Q. Unraveling EGFR-TKI resistance in lung cancer with high PD-L1 or TMB in EGFR-sensitive mutations. Respir Res. (2024) 25:40. 38238740 10.1186/s12931-023-02656-3 PMC10797755

[14] Bhardwaj N Rohilla M Trehan A Bansal D Kakkar N Srinivasan R. MYCN amplification and International Neuroblastoma Risk Group stratification on fine-needle aspiration biopsy and their correlation to survival in neuroblastoma. J Clin Pathol. (2023) 76:599–605. 35414524 10.1136/jclinpath-2022-208177

[15] Chen ZY Duan XT Qiao SM Zhu XX. Radiotherapy combined with PD-1/PD-L1 inhibitors in NSCLC brain metastases treatment: The mechanisms, advances, opportunities, and challenges. Cancer Med. (2023) 12:995–1006., PMID: 35986515 10.1002/cam4.5016 PMC9883424

[16] Tawbi HA Forsyth PA Hodi FS Algazi AP Hamid O Lao CD. Long-term outcomes of patients with active melanoma brain metastases treated with combination nivolumab plus ipilimumab (CheckMate 204): final results of an open-label, multicentre, phase 2 study. Lancet Oncol. (2021) 22:1692–704., PMID: 34774225 10.1016/S1470-2045(21)00545-3 PMC9328029

[17] Tawbi HA Forsyth PA Algazi A Hamid O Hodi FS Moschos SJ. Combined nivolumab and ipilimumab in melanoma metastatic to the brain. N Engl J Med. (2018) 379:722–30., PMID: 30134131 10.1056/NEJMoa1805453 PMC8011001

[18] Nguyen B Fong C Luthra A Smith SA DiNatale RG Nandakumar S. Genomic characterization of metastatic patterns from prospective clinical sequencing of 25,000 patients. Cell. (2022) 185:563–575.e511., PMID: 35120664 10.1016/j.cell.2022.01.003 PMC9147702

[19] Zehir A Benayed R Shah RH Syed A Middha S Kim HR. Mutational landscape of metastatic cancer revealed from prospective clinical sequencing of 10,000 patients. Nat Med. (2017) 23:703–13. 10.1038/nm.4333 PMC5461196 28481359

[20] Jee J Fong C Pichotta K Tran TN Luthra A Waters M. Automated real-world data integration improves cancer outcome prediction. Nature. (2024) 636:728–36., PMID: 39506116 10.1038/s41586-024-08167-5 PMC11655358

[21] Cheng DT Mitchell TN Zehir A Shah RH Benayed R Syed A. Memorial sloan kettering-Integrated mutation profiling of actionable cancer targets (MSK-IMPACT): A hybridization capture-Based next-Generation sequencing clinical assay for solid tumor molecular oncology. J Mol Diagn. (2015) 17:251–64. 10.1016/j.jmoldx.2014.12.006 PMC5808190 25801821

[22] Ramos AH Lichtenstein L Gupta M Lawrence MS Pugh TJ Saksena G. Oncotator: cancer variant annotation tool. Hum Mutat. (2015) 36:E2423–2429., PMID: 25703262 10.1002/humu.22771 PMC7350419

[23] Rizvi NA Hellmann MD Snyder A Kvistborg P Makarov V Havel JJ. Cancer immunology. Mutational landscape determines sensitivity to PD-1 blockade in non-small cell lung cancer. Science. (2015) 348:124–8., PMID: 25765070 10.1126/science.aaa1348 PMC4993154

[24] Hellmann MD Nathanson T Rizvi H Creelan BC Sanchez-Vega F Ahuja A. Genomic features of response to combination immunotherapy in patients with advanced non-Small-Cell lung cancer. Cancer Cell. (2018) 33:843–852.e844. 29657128 10.1016/j.ccell.2018.03.018 PMC5953836

[25] Snyder A Makarov V Merghoub T Yuan J Zaretsky JM Desrichard A. Genetic basis for clinical response to CTLA-4 blockade in melanoma. N Engl J Med. (2014) 371:2189–99. 10.1056/NEJMoa1406498 PMC4315319 25409260

[26] Van Allen EM Miao D Schilling B Shukla SA Blank C Zimmer L. Genomic correlates of response to CTLA-4 blockade in metastatic melanoma. Science. (2015) 350:207–11. 10.1126/science.aad0095 PMC5054517 26359337

[27] Hugo W Zaretsky JM Sun L Song C Moreno BH Hu-Lieskovan S. Genomic and transcriptomic features of response to anti-PD-1 therapy in metastatic melanoma. Cell. (2016) 165:35–44. 26997480 10.1016/j.cell.2016.02.065 PMC4808437

[28] Zaretsky JM Garcia-Diaz A Shin DS Escuin-Ordinas H Hugo W Hu-Lieskovan S. Mutations associated with acquired resistance to PD-1 blockade in melanoma. N Engl J Med. (2016) 375:819–29., PMID: 27433843 10.1056/NEJMoa1604958 PMC5007206

[29] Riaz N Havel JJ Makarov V Desrichard A Urba WJ Sims JS. Tumor and microenvironment evolution during immunotherapy with nivolumab. Cell. (2017) 171:934–949.e916., PMID: 29033130 10.1016/j.cell.2017.09.028 PMC5685550

[30] Roh W Chen PL Reuben A Spencer CN Prieto PA Miller JP. Integrated molecular analysis of tumor biopsies on sequential CTLA-4 and PD-1 blockade reveals markers of response and resistance. Sci Transl Med. (2017) 9:eaah3560. 28251903 10.1126/scitranslmed.aah3560 PMC5819607

[31] Liu D Schilling B Liu D Sucker A Livingstone E Jerby-Arnon L. Integrative molecular and clinical modeling of clinical outcomes to PD1 blockade in patients with metastatic melanoma. Nat Med. (2019) 25:1916–27. 10.1038/s41591-019-0654-5 PMC6898788 31792460

[32] Devarajan K. Nonnegative matrix factorization: an analytical and interpretive tool in computational biology. PLoS Comput Biol. (2008) 4:e1000029., PMID: 18654623 10.1371/journal.pcbi.1000029 PMC2447881

[33] Mayakonda A Lin DC Assenov Y Plass C Koeffler HP. Maftools: efficient and comprehensive analysis of somatic variants in cancer. Genome Res. (2018) 28:1747–56., PMID: 30341162 10.1101/gr.239244.118 PMC6211645

[34] Tate JG Bamford S Jubb HC Sondka Z Beare DM Bindal N. COSMIC: the catalogue of somatic mutations in cancer. Nucleic Acids Res. (2019) 47:D941–7. 10.1093/nar/gky1015 PMC6323903 30371878

[35] Alexandrov LB Nik-Zainal S Wedge DC Aparicio SA Behjati S Biankin AV. Signatures of mutational processes in human cancer. Nature. (2013) 500:415–21., PMID: 23945592 10.1038/nature12477 PMC3776390

[36] Wilkerson MD Hayes DN. ConsensusClusterPlus: a class discovery tool with confidence assessments and item tracking. Bioinformatics. (2010) 26:1572–3., PMID: 20427518 10.1093/bioinformatics/btq170 PMC2881355

[37] Gu Z. Complex heatmap visualization. Imeta. (2022) 1:e43. 38868715 10.1002/imt2.43 PMC10989952

[38] Jardim DL Goodman A de Melo Gagliato D Kurzrock R. The challenges of tumor mutational burden as an immunotherapy biomarker. Cancer Cell. (2021) 39:154–73., PMID: 33125859 10.1016/j.ccell.2020.10.001 PMC7878292

[39] Yarchoan M Hopkins A Jaffee EM. Tumor mutational burden and response rate to PD-1 inhibition. N Engl J Med. (2017) 377:2500–1., PMID: 29262275 10.1056/NEJMc1713444 PMC6549688

[40] Le DT Durham JN Smith KN Wang H Bartlett BR Aulakh LK. Mismatch repair deficiency predicts response of solid tumors to PD-1 blockade. Science. (2017) 357:409–13. 10.1126/science.aan6733 PMC5576142 28596308

[41] Jiang M Jia K Wang L Li W Chen B Liu Y. Alterations of DNA damage response pathway: Biomarker and therapeutic strategy for cancer immunotherapy. Acta Pharm Sin B. (2021) 11:2983–94. 10.1016/j.apsb.2021.01.003 PMC8546664 34729299

[42] Samstein RM Lee CH Shoushtari AN Hellmann MD Shen R Janjigian YY. Tumor mutational load predicts survival after immunotherapy across multiple cancer types. Nat Genet. (2019) 51:202–6., PMID: 30643254 10.1038/s41588-018-0312-8 PMC6365097

[43] McNamara MG Jacobs T Lamarca A Hubner RA Valle JW Amir E. Impact of high tumor mutational burden in solid tumors and challenges for biomarker application. Cancer Treat Rev. (2020) 89:102084., PMID: 32738738 10.1016/j.ctrv.2020.102084

[44] Hodis E Watson IR Kryukov GV Arold ST Imielinski M Theurillat JP. A landscape of driver mutations in melanoma. Cell. (2012) 150:251–63., PMID: 22817889 10.1016/j.cell.2012.06.024 PMC3600117

[45] Zhang X Guo A Yu J Possemato A Chen Y Zheng W. Identification of STAT3 as a substrate of receptor protein tyrosine phosphatase T. Proc Natl Acad Sci U.S.A. (2007) 104:4060–4., PMID: 17360477 10.1073/pnas.0611665104 PMC1802729

[46] Kim M Morales LD Jang IS Cho YY Kim DJ. Protein tyrosine phosphatases as potential regulators of STAT3 signaling. Int J Mol Sci. (2018) 19:2708. 10.3390/ijms19092708 PMC6164089 30208623

[47] Ou C Peng Q Zeng C. An integrative prognostic and immune analysis of PTPRD in cancer. Math Biosci Eng. (2022) 19:5361–79., PMID: 35603359 10.3934/mbe.2022251

[48] Shang X Zhang W Zhang X Yu M Liu J Cheng Y. PTPRD/PTPRT mutation as a predictive biomarker of immune checkpoint inhibitors across multiple cancer types. Front Immunol. (2022) 13:991091., PMID: 36248841 10.3389/fimmu.2022.991091 PMC9556668

[49] Carnero A Blanco-Aparicio C Renner O Link W Leal JF. The PTEN/PI3K/AKT signalling pathway in cancer, therapeutic implications. Curr Cancer Drug Targets. (2008) 8:187–98., PMID: 18473732 10.2174/156800908784293659

[50] Pan AL Audrain M Sakakibara E Joshi R Zhu X Wang Q. Dual-Specificity protein phosphatase 4 (DUSP4) overexpression improves learning behavior selectively in female 5xFAD mice, and reduces beta-Amyloid load in males and females. Cells. (2022) 11:3880., PMID: 36497141 10.3390/cells11233880 PMC9737364

[51] Toth AE Helms HC Harazin A Johnsen KB Goldeman C Burkhart A. Sortilin regulates blood-brain barrier integrity. FEBS J. (2022) 289:1062–79., PMID: 34626084 10.1111/febs.16225

[52] Wang LF Li X Gao YB Wang SM Zhao L Dong J. Activation of VEGF/Flk-1-ERK pathway induced blood-Brain barrier injury after microwave exposure. Mol Neurobiol. (2015) 52:478–91., PMID: 25195697 10.1007/s12035-014-8848-9

[53] Heydt C Wolwer CB Velazquez Camacho O Wagener-Ryczek S Pappesch R Siemanowski J. Detection of gene fusions using targeted next-generation sequencing: a comparative evaluation. BMC Med Genomics. (2021) 14:62., PMID: 33639937 10.1186/s12920-021-00909-y PMC7912891

[54] Lord CJ Ashworth A. PARP inhibitors: Synthetic lethality in the clinic. Science. (2017) 355:1152–8. 10.1126/science.aam7344 PMC6175050 28302823

