# Supplementary material for: A mutational process signature and genomic alterations associated with outcome and immunogenicity in cancers with brain metastasis
Source: Front Immunol. 2025 Jul 30;16:1607772. doi: 10.3389/fimmu.2025.1607772 (PMC12343737; doi:10.3389/fimmu.2025.1607772)
Supplement: Supplementary file 1 [file DataSheet1.docx]

**Supplementary Figures
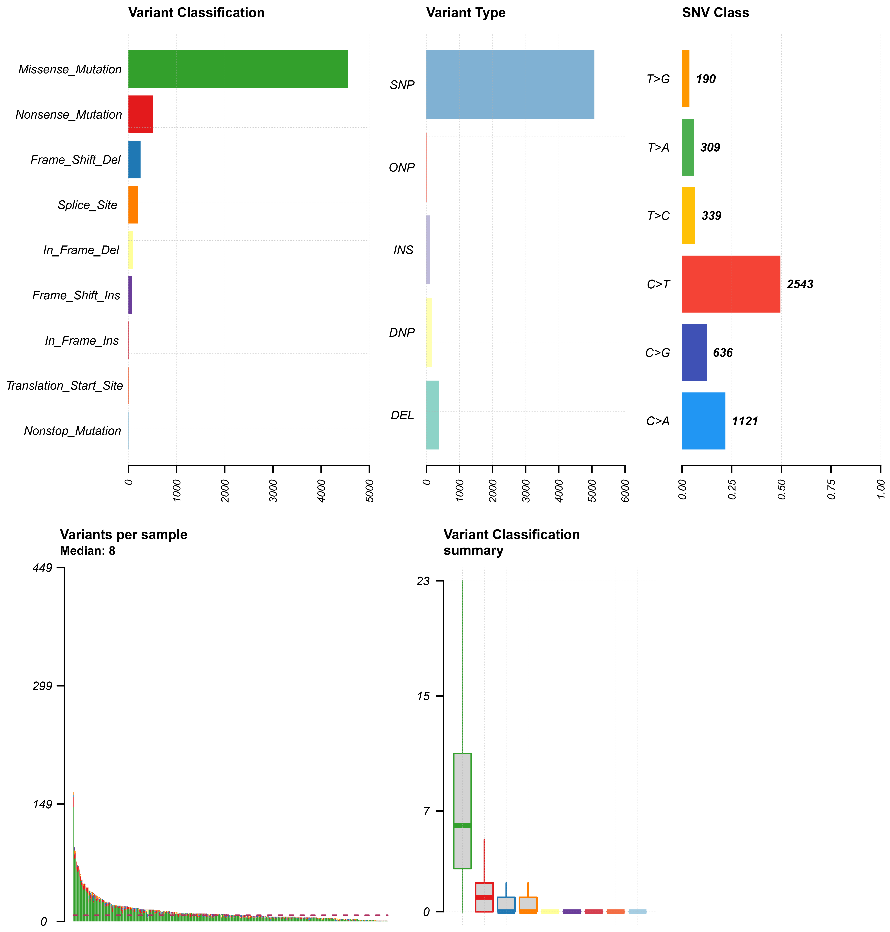
**

**
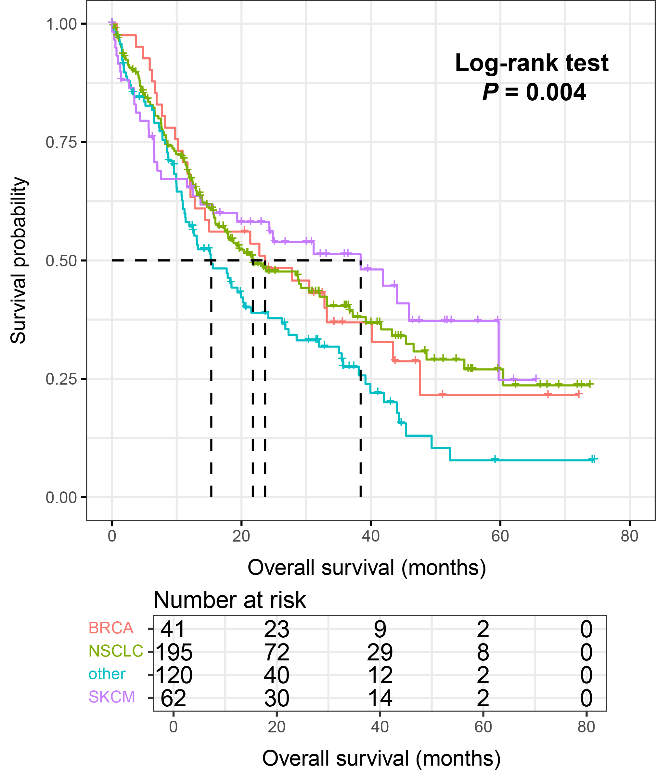
Figure S1. Detailed mutational characteristics of the 421 BM tumor patients included in this study.**

**Figure S2. Kaplan-Meier survival curves of different primary tumor types of BM (NSCLC, SKCM, BRCA and other types).**

**
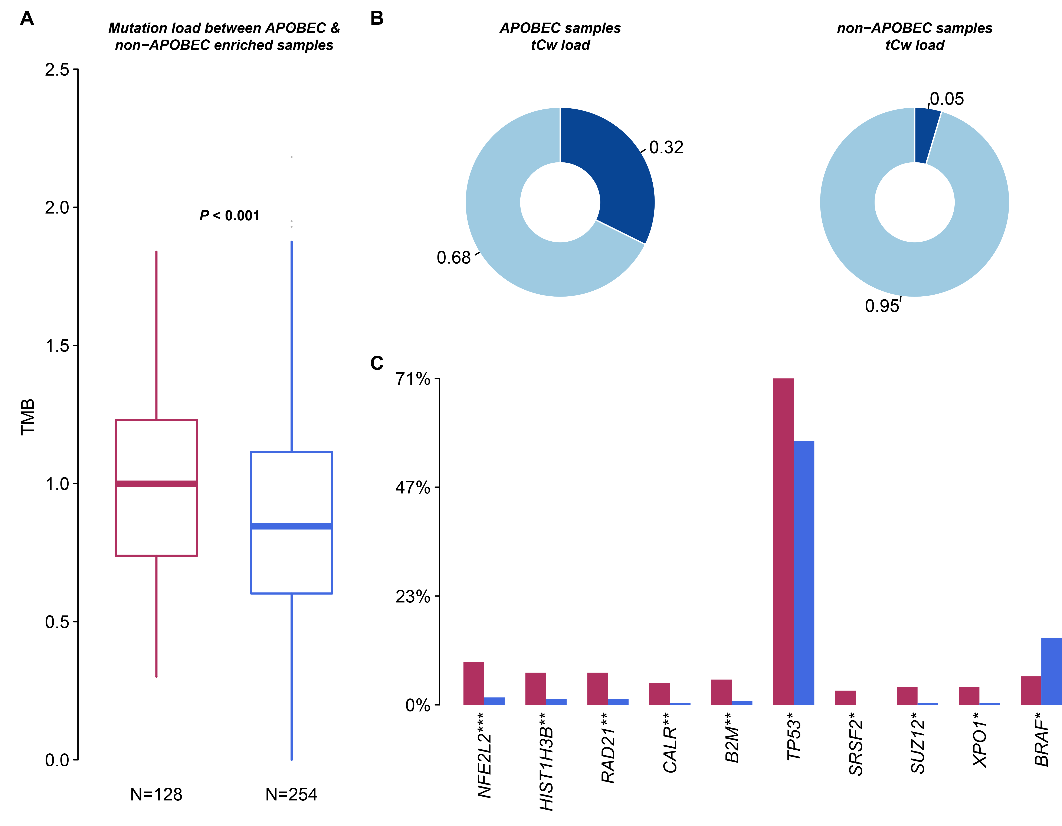
**

**Figure S3. APOBEC-related mutaitonal process signature operative in BM patients.** (A) The difference in TMB levels between BM patients with and without the APOBEC mutation signature. (B) The difference in tCw burden between BM patients with and without the APOBEC mutation signature. (C) Genes with significantly different mutation frequencies between BM patients with and without the APOBEC mutation signature. * *P* < 0.05, ** *P* < 0.01, *** *P* < 0.001

**
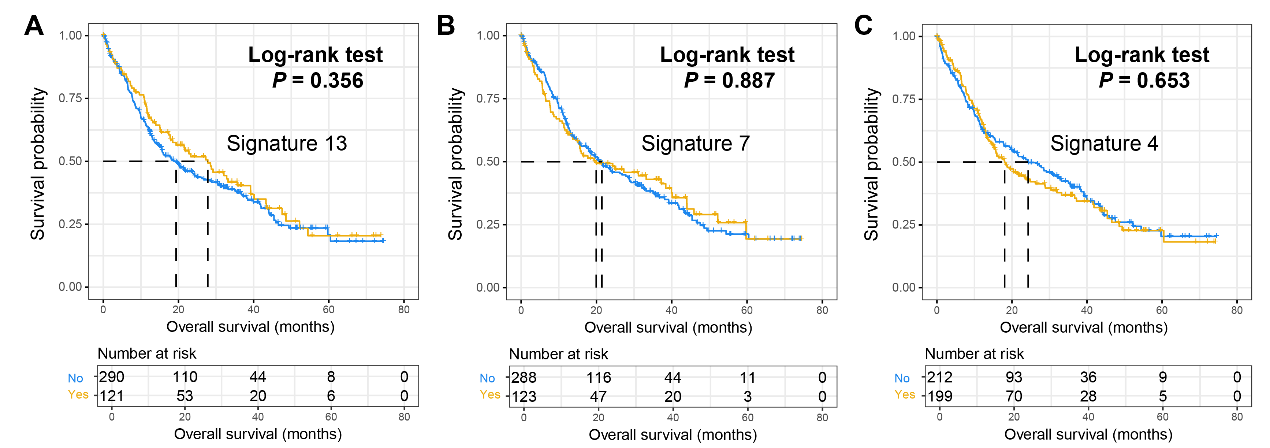
**

**Figure S4. Survival curves of BM patients with and without the mutational signatures of (A) signature 13, (B) signature 7, and (C) signature 4.**

**Figure S
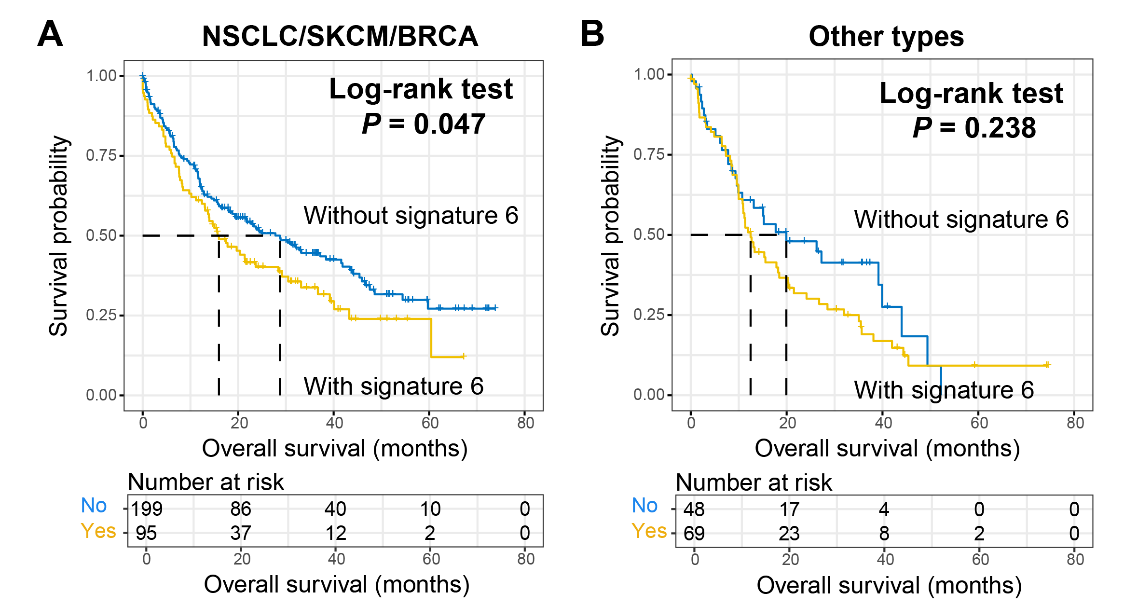
5. Kaplan-Meier survival curves for patients with and without signature 6 in primary tumors of BM patients: (A) NSCLC/SKCM/BRCA and (B) other tumor types.**

**
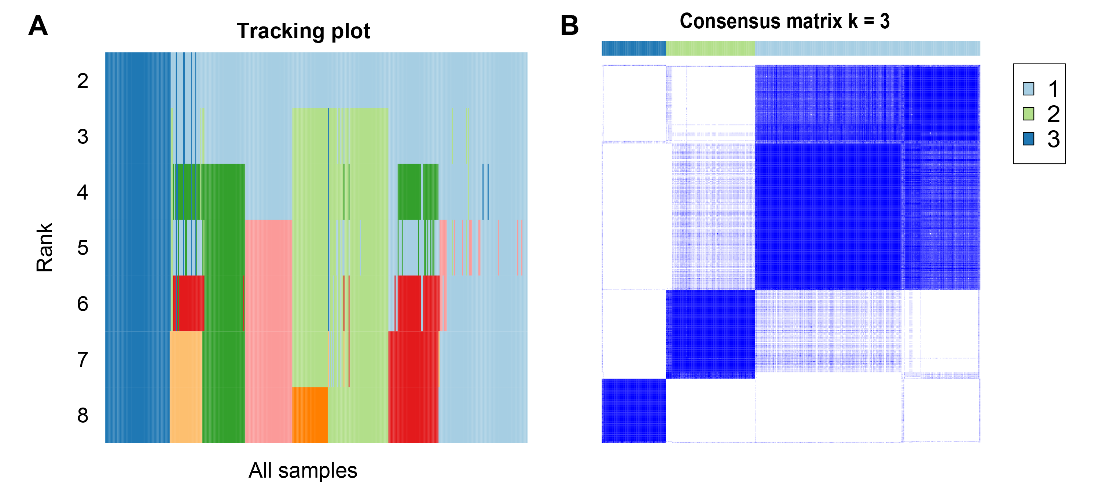
**

**Figure S6. (A) The clustering tracking plot when the number of clusters based on the activity of mutation signatures ranged from 2 to 8. (B) The clustering heatmap of BM patients when the number of clusters was selected as 3.**

**
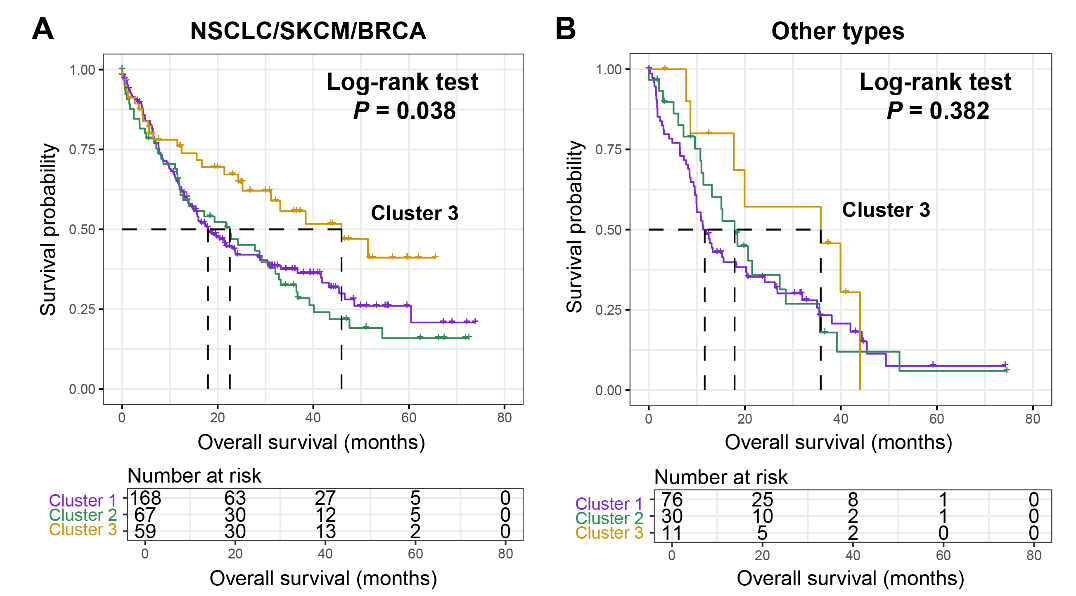
Figure S7. Kaplan-Meier survival curves for patients of identified three BM clusters in primary tumors of BM patients: (A) NSCLC/SKCM/BRCA and (B) other tumor types.**

**
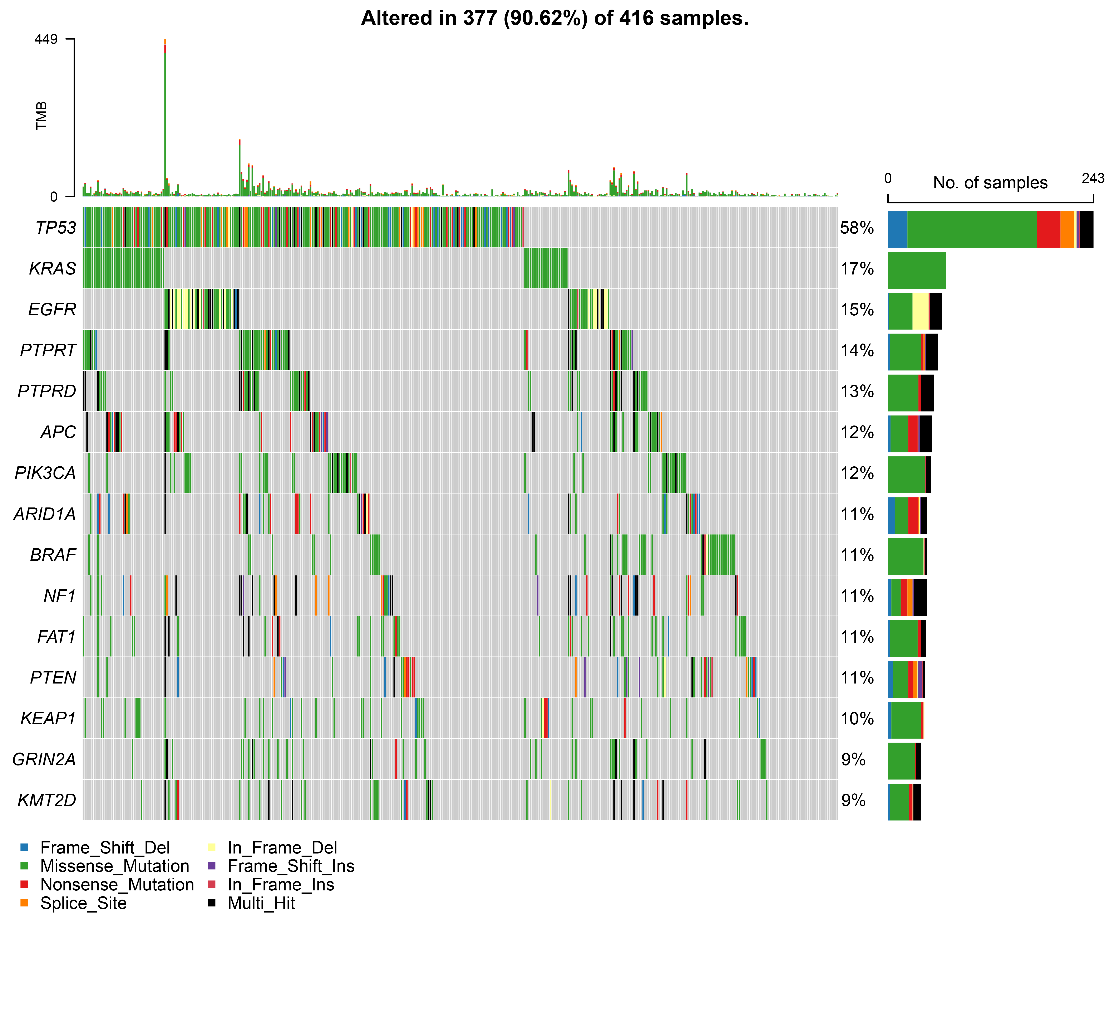
**

**Figure S8.** **A waterfall plot showing the commonly recurrently mutated genes in BM patients.**

**
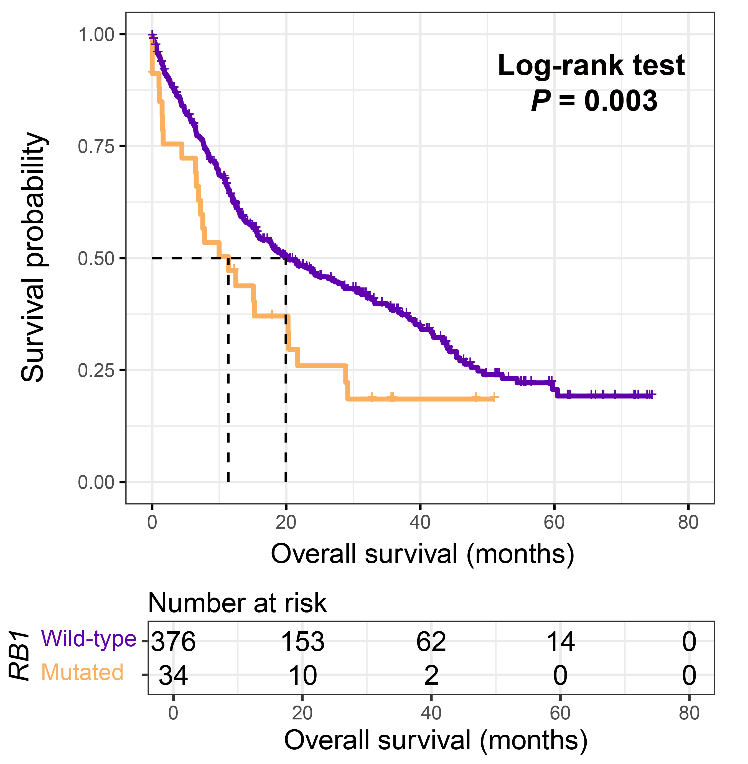
**

**
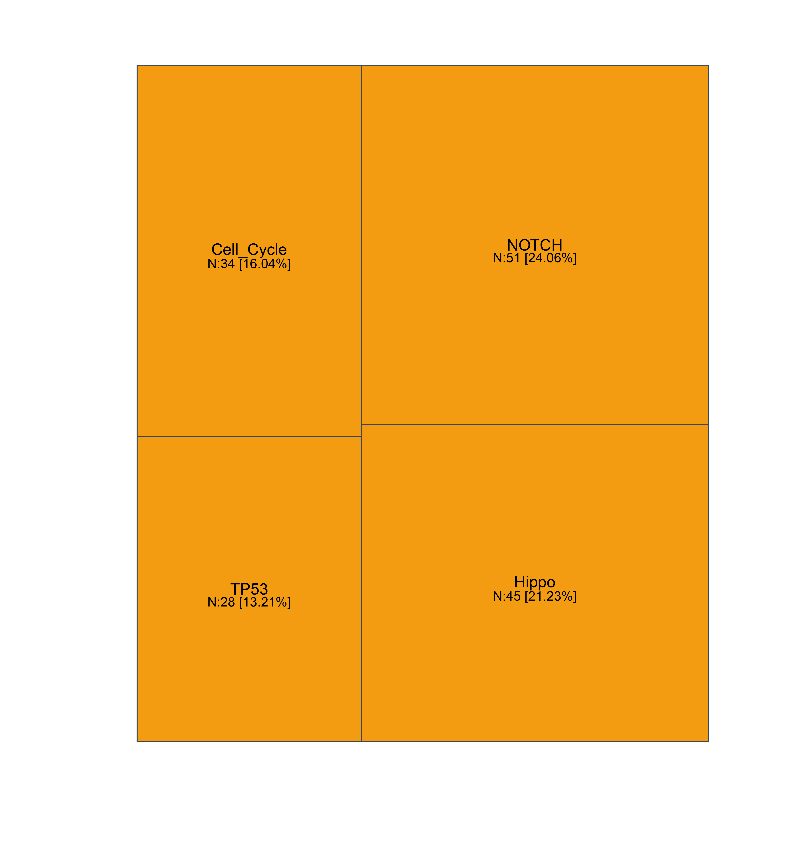
Figure S9.** **Kaplan-Meier survival curves stratified by *RB1* mutation and wild-type BM patients.**

**Figure S10.** **The signaling pathways significantly enriched by the recurrently prognostically mutated genes in BM patients.**

**
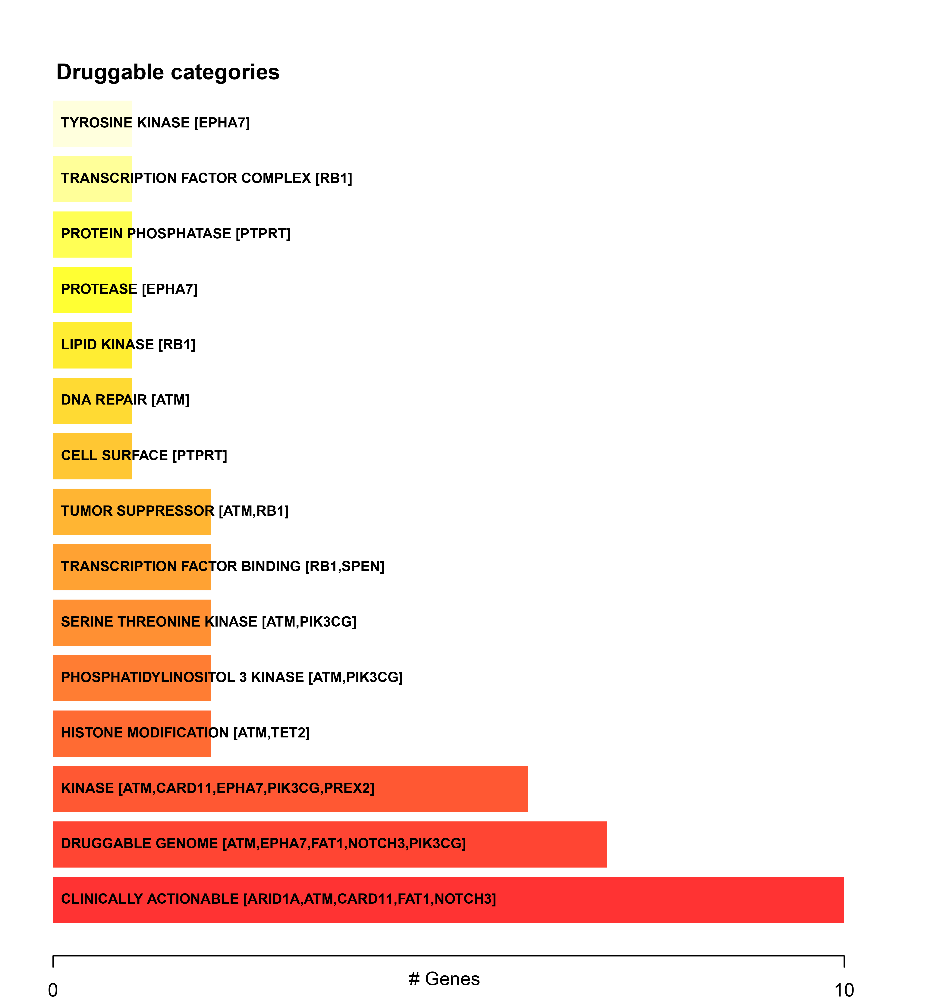
Figure S11.** **Potential therapeutic targetability of BM prognostically mutated genes stratified by druggable categories.**
